# Increased Prolylcarboxypeptidase Expression Can Serve as a Biomarker of Senescence in Culture

**DOI:** 10.3390/molecules29102219

**Published:** 2024-05-09

**Authors:** Nicholas Glen Boullard, Jason J. Paris, Zia Shariat-Madar, Fakhri Mahdi

**Affiliations:** 1 Jackson Hinds Comprehensive Health Center, Jackson, MS 39216, USA; msmca.gluckstadt@gmail.com; 2 Division of Pharmacology, School of Pharmacy, University of Mississippi, Oxford, MS 38677, USA; parisj@olemiss.edu (J.J.P.); madar@olemiss.edu (Z.S.-M.)

**Keywords:** cardiovascular dysfunction, metabolic syndrome, renin–angiotensin system, kallikrein–kinin system, human telomerase reverse transcriptase, complex I inhibitor

## Abstract

Prolylcarboxypeptidase (PRCP, PCP, Lysosomal Pro-X-carboxypeptidase, Angiotensinase C) controls angiotensin- and kinin-induced cell signaling. Elevation of PRCP appears to be activated in chronic inflammatory diseases [cardiovascular disease (CVD), diabetes] in proportion to severity. Vascular endothelial cell senescence and mitochondrial dysfunction have consistently been shown in models of CVD in aging. Cellular senescence, a driver of age-related dysfunction, can differentially alter the expression of lysosomal enzymes due to lysosomal membrane permeability. There is a lack of data demonstrating the effect of age-related dysfunction on the expression and function of PRCP. To explore the changes in PRCP, the PRCP-dependent prekallikrein (PK) pathway was characterized in early- and late-passage human pulmonary artery endothelial cells (HPAECs). Detailed kinetic analysis of cells treated with high molecular weight kininogen (HK), a precursor of bradykinin (BK), and PK revealed a mechanism by which senescent HPAECs activate the generation of kallikrein upon the assembly of the HK–PK complex on HPAECs in parallel with an upregulation of PRCP and endothelial nitric oxide (NO) synthase (eNOS) and NO formation. The NO production and expression of both PRCP and eNOS increased in early-passage HPAECs and decreased in late-passage HPAECs. Low activity of PRCP in late-passage HPAECs was associated with rapid decreased telomerase reverse transcriptase mRNA levels. We also found that, with an increase in the passage number of HPAECs, reduced PRCP altered the respiration rate. These results indicated that aging dysregulates PRCP protein expression, and further studies will shed light into the complexity of the PRCP-dependent signaling pathway in aging.

## 1. Introduction

As we age, all of our biological processes and functions lose their efficiency. Age is not a direct cause of death, but the decreased function of our bodies, brought on by age, is. It has been long recognized that aging is as a major risk factor for numerous diseases including inflammation, metabolic syndrome, and CVD. Leonard Hayflick [1] observed that a culture of human tissue, in vitro, stopped dividing once the culture had reached a fixed threshold age via numerous cellular divisions. It was hypothesized that this phenomenon could be used to study human aging at the cellular level [2]. 

Endothelial metabolic changes associated with aging have been linked to vascular remodeling in blood vessels [3] and decline in blood flow and increased fluid shear stress, which become the driving force in activating platelets and inducing thrombosis. Aging-induced reduction in microcirculation plasticity and aging-associated telomeric DNA damage contribute to the pathogenesis of the range of age-related diseases, including those affecting the brain [4], heart [5], kidney [6], and eyes. The general consensus is that aging is driven largely by reactive oxygen species (ROS) produced in mitochondria, which can promote oxidative damage to the mitochondrial protein, membrane, and DNA. ROS has been documented to damage telomeric DNA effectively [7,8]. ROS also causes lipid peroxidation [9] and promotes a prothrombotic state in the vascular system. Hypertension, a limb of the metabolic syndrome, is associated with diabetes and impaired glucose tolerance. Notably, high glucose concentrations are associated with elevated ROS and reduced NO, highlighting the presence of a bidirectional response [10]. These studies support the conclusion that aging endothelial cells undergo metabolic changes and are simply unable to maintain antioxidant–ROS balance. Moreover, the aging of cells could be directly linked to genomic DNA [11] or environmental factors (such as salt intake and medications) that alter the production of angiotensin II (Ang II, a potent vasoconstrictor) [12].

It has been suggested that endothelial cell replicative senescent is a feature of type 2 diabetes mellitus (T2DM) and atherosclerosis. Although endothelial dysfunction is recognized as an initial step in atherosclerotic vascular disease, it is advanced in diabetes. PRCP is expressed in endothelial cells. All arteries, veins, and capillaries of the human circulatory system produce PRCP. Plasma PRCP was elevated in diabetic patients [13] while its substrate plasma kallikrein (the G allele of *KLKB1* rs3733402) activity correlated with a reduced history of CVD [14]. PRCP plays a role in regulating the function of activated cells in restoring and maintaining cellular homeostasis. For instance, PRCP metabolizes Ang II [15] to angiotensin (1–7) (Ang_1–7,_ a vasodilator) and angiotensin III (Ang III, a vasoconstrictor) [16] to angiotensin (2–7) (Ang 2–7) at acidic pH [17]. Recent evidence indicates that PRCP is capable of protecting the heart from Ang II-induced hypertrophic remodeling via controlling myocardial Ang II levels [18]. Bradykinin protects endothelial cells. PRCP also activates the plasma PK to kallikrein in the presence of HK [19]. Formed kallikrein cleaves HK to liberate BK [20]. Activation of the BKB2 (B_2_) receptors [21] by BK and the Mas oncogene receptors [22] by Ang1–7 lead, among others, to vasorelaxation and improving cell metabolism via the generation of nitric oxide (NO), which was found to prevent endothelial senescence [23]. Strikingly, it is controversial as to whether Ang 1–7 mediates its effect via the proto-oncogene Mas receptors [24]. Regardless of the binding of the Ang 1–7 to Mas receptors, PRCP is capable of inactivating both Ang II and Ang III, both of which play crucial roles not only in the release of aldosterone from the adrenal glands, but also in the modulation of vascular tone. Thus, PRCP backs the endothelium-dependent relaxation. 

PRCP expression has been found to be altered under pathologic conditions of inflammation, hyperlipidemia, diabetes, obesity, and hypertension. The central issue of PRCP involvement in the pathogenesis of experimental hypertension and heart transplant patients was defined as an essential identity for both renal and cardiac Ang1–7 formation [25]. Wu et al. [26] observed susceptibility for hypertension in Han Chinese individuals without history of diabetes mellitus (DM) with the G allele of *PRCP* SNP rs7104980. Another interesting observation suggests that this SNP of *PRCP* may be a potential cardiovascular risk factor for percutaneous transluminal coronary angioplasty (PTCA) [14]. A decrease in PRCP level was reported following chronic ethanol regimen in spontaneously hypertensive rats (SHRs) [27]. Furthermore, their findings also indicated that the downregulation of PRCP in addition to enhanced RAS activity may provoke further deterioration of left ventricular (LV) systolic dysfunctions in SHRs. While the PRCP gene variant affects the progression of hypertension [28], its depletion results in vascular dysfunction and faster arterial thrombosis in mice [29]. Endothelial dysfunction is induced in hypertensive patients [9]. Additional studies are required to confirm whether PRCP plays an essential role in endothelial cell regulation.

Here, we aimed to examine (1) whether senescent HPAECs are predisposed to enhanced PK activation due to enhanced HK binding, (2) whether senescent HPAECs express lower levels of PRCP mRNA, protein, and activity and produce less NO compared with early-passage cells, and (3) whether the inhibition of PRCP is associated with the disruption of mitochondrial bioenergetics in HPAECs.

## 2. Results

### 2.1. Effect of Aging on Kallikrein Activity in an HPAEC Line

**Figure 1:**
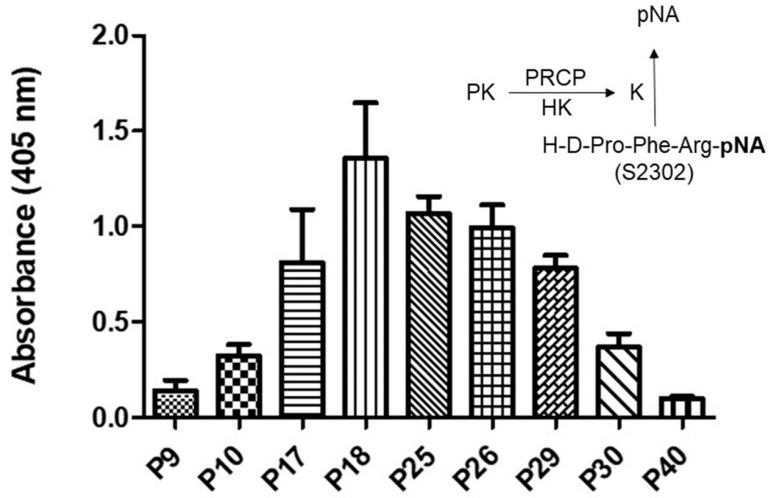
Plasma kallikrein activity in various HPAE cell passages. A total of 40,000 HPAE cells/well were cultured in a 96-well plate for a total of 17 to 18 h. An amount of 20 nM PK was incubated with 20 nM HK bound to HPAE cells at 37 °C. The liberation of paranitroaniline (pNA) from chromogenic substrate (S2302) by kallikrein was measured at 405 nm. Inset, endothelial (HPACs) PRCP activates PK (a zymogen) to kallikrein, which leads to the release of paranitroaniline (pNA) from S2302. All values are expressed as mean + SEM of triplicate points of and represent at least three independent experiments.

Evidence indicates that the binding of PK to HK bound to HPAECs lead to its activation to kallikrein via PRCP [30]. Here, we examined whether PRCP-dependent PK activation is altered in an HPAEC line at various passages (Figure 1). Since there was no defined working passage (subculture) number for the HPAEC line, we investigated PK activation from the complex of HK and PK in working passages (P3–P20) and late passages (P21–P40). HPAECs were grown as previously described [31]. Briefly, monolayers of HPAECs (80–90% confluence) were washed three times with HEPES–NaHCO_3_ buffer and then blocked with 1% gelatin for 1 h at 37 °C. After washing, 20 nM HK was added to HPAECs and incubated for additional 1 h at 37 °C. At the end of the incubation, 20 nM PK was added and incubated for 1 h at 37 °C. Kallikrein activity was detected by the absorbance of freed paranitoaniline (pNA) from chromogenic substrate S2302. Amidolytic activity was measured in 100 μL of assay buffer. Kallikrein activity was observed in all cell passages (Figure 1). 

Kallikrein activity progressively increased from P7 to P19, peaking in working passages (P17–P19), and then a gradual decline in enzyme activity was observed in late passages (P21–P40) (Figure 1). Together, these data demonstrated that PK activation is cell passage-dependent, and late-culture cell passage experience alters the response to stimuli such as the HK–PK complex.

### 2.2. Correlation between PRCP Expression and Kallikrein Activity in HPAECs at Various Passages

**Figure 2:**
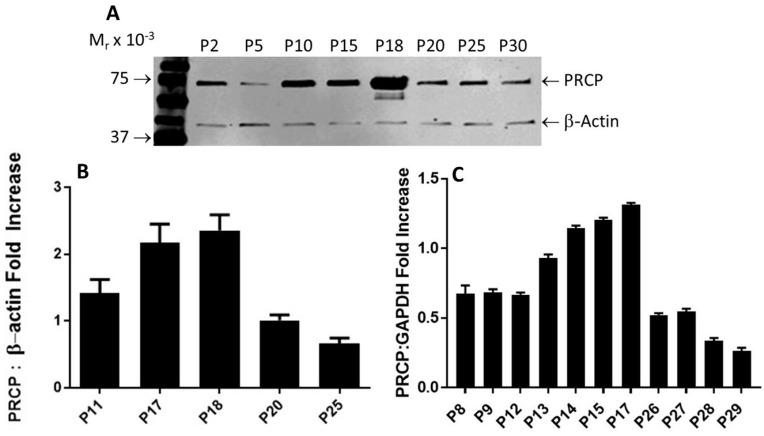
PRCP expression is age-dependent. (**A**), Representative Western blots of PRCP protein expression of whole-cell lysates from both working passages and late passages (P2–P30). (**B**), Densitometric analysis of relative density from three independent Western blot experiments. Bars display mean + SEM. (**C**), Densitometric analysis of RT-PCR of PRCP in cultured endothelial cells from HPAECs of both working and late passages of three independent total RNA preparations. Total RNA was isolated from HPAECs and then amplified by RT-PCR. Amplified DNA (111 bp fragment) was resolved on 1.5% agarose gel. Values are expressed as mean + SEM of triplicate points of 3 independent experiments.

PRCP-dependent PK activation is evident in endothelial cells [32]. It appears to play a direct role in mediating vasodilation through the bradykinin B_2_ pathway and opposing Ang II-mediated vasoconstriction via AT2R, helping maintain vascular endothelial integrity. Since PK activation peaked in working passages (P17–P19), PRCP mRNA and protein expression were measured and compared to those of lower and higher passages as shown in Figure 2. 

After performing multiple Western blots to measure the protein expression of PRCP, a trend similar to that observed for HK–PK activity was found. PRCP protein levels increased from passages 10 to 18, followed by a significant decrease in late passages (P20–P30) (Figure 2A). β-actin served as a loading control (Figure 2A). Original blot can be found in Supplementary Materials Figure S1. Densitometric analysis of bands showed a 40% to 65% reduction in PRCP expression in the late passage (Figure 2B). Next, we investigated PRCP mRNA expression in resting cells at passages (P8–P29). We observed an increase of PRCP mRNA from working passages 8 through 17 even though the endothelial cells of passages 8, 9, and 12 displayed unaltered expression of PRCP. However, a gradual decrease in PRCP mRNA expression was observed in late passages (P22–P29). As a control, glyceraldehyde-3-phosphate dehydrogenase (GAPDH) was used. These changes mirrored PRCP protein levels (Figure 2C). A strong signal for PRCP mRNA was observed in working passages compared to late passages. PRCP production in late passages also was significantly lower than that of the working passages. These data demonstrate a role for PRCP in regulating PK in endothelial cells. 

### 2.3. PRCP Delays Cellular Senescence through an NO-Dependent Mechanism

**Figure 3:**
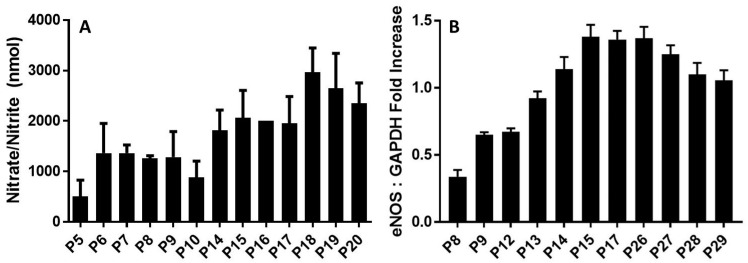
eNOS plays a role in maintaining endothelial integrity during aging. (**A**), NO production by HPAECs. The kallikrein–kinin generating pathway induces nitric oxide (NO) generation in a time-dependent manner. Both working passages (P9, P10, P17, P18)- and late passages (P25, P26, P29, P30, P40)- HPAE cells were incubated with 300 nM HK and 300 nM PK for 17 h at 37 °C. The solution was collected to measure the amount of nitrate+nitrite (the final products of NO metabolism) in each sample using a fluorometric assay. Data are presented as mean + SEM (*n* = 3). (**B**), Densitometric analysis of RT-PCR of eNOS mRNA in cultured endothelial cells from HPAECs of both working and late passages of three independent total RNA preparations. Values are expressed as mean ± SEM of three independent experiments.

Prior studies have shown that the activation of PK to kallikrein by PRCP leads to the liberation of BK [33]. Formed BK then binds to B_2_ receptors on the endothelial cell surface to release NO and prostaglandin I_2_ (PGI, prostacyclin) [34]. The assembly of the complex of HK and PK on HPAECs induced NO formation in the working passages (P5–P19) (Figure 3A). However, there was a decline in NO production from the late passage (P20). Understandably, as the cell passage increased, so did the amount of NO products. This supports the idea that the effects of the HK–PK complex on the endothelium are important for understanding the pathophysiology of aging.

Next, we evaluated changes in the expression levels of endothelial nitric oxide synthase (eNOS). The relative levels of eNOS mRNA significantly changed during cell aging (Figure 3B). GAPDH served as a positive control. After cell passage 8, the expression of eNOS of passages (P9–P26) was increased markedly upon binding of the HK–PK complex to cells, to a maximum of three times more than that of passage 8. After passage 26, eNOS expression levels gradually declined. This slow decrease is due to the rising need for NO to oxidize the increased quantity of reactive oxygen species (ROS) when enough substrate L-arginine and cofactor BH4 are present, or due to increased production of superoxide (O_2•_^−^) by eNOS (referred to as eNOS uncoupling) [35]. As NO is rapidly used up, eNOS remains steady for several late, senescent passages. Our results supported previous findings with NO in aging cells [36], showing that there is also a rapid increase in eNOS over time, followed by a slow decrease. These results indicated that the upregulation of eNOS mRNA was consistent with the increase in kallikrein formation (Figure 1) and elevation of PRCP enzyme activity (Figure 2), suggesting that PRCP might play a key role in protecting cell survival via scavenging ROS.

### 2.4. β-Galactosidase Activity Increases with Age

**Figure 4:**
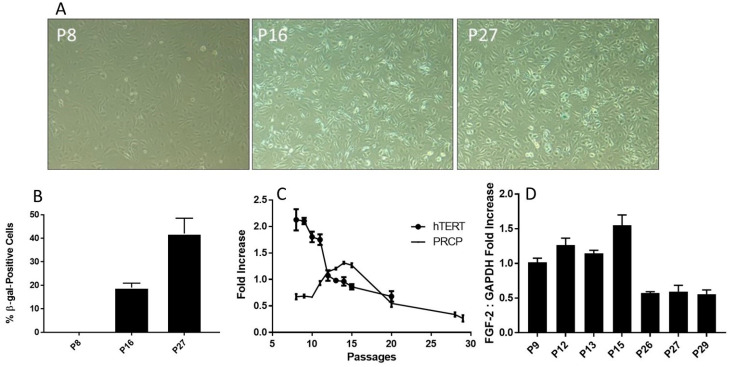
Senescence and telomere length. (**A**), Representative photographs of β-gal-positive staining cells. The cells were examined on a light microscope with an attached camera using a 10× objective. The β-gal-positive cells were blue. Representative images were used for figures. Positive staining cells were determined (*n* = 3). (**B**), The relative levels of β-gal-positive staining in various HPAEC passages. The fraction of β-gal-positive cells was determined by counting the number of blue cells in 5 non-overlapping images from various fields of view within each well. The β-gal-positive (blue) cell count was divided by the number of total cell count to calculate the percentage senescent cells. Graph shows mean data ± SEM from three experiments, with at least 500 cells counted per condition per experiment. (**C**), Reduced transcription of telomerase (hTERT) mRNA. HPAECs were continuously passaged, and hTERT mRNA was determined. The decrease in telomerase was accompanied by an increase in PRCP at working passages (P5–P20). (**D**), The effect of aging on the level of FGF-2 expression. HPAECs were passaged, and FGF-2 expression was determined. Each bar displays mean data + SEM from three experiments.

In order to better visualize the effect of aging on endothelial cell senescence, β-galactosidase (β-gal), a marker protein for senescence, staining was implemented on various HPAEC passages. Using light microscopy, we observed an increase in β-gal-positive cells (Figure 4A), confirming a previously published work [37]. The fraction of β-gal-positive cells was gradually increased from P16, reaching its maximum at late passage (P27). Relative cell count was measured and graphed (Figure 4B). β-gal concentration became apparent at passage P16 (at peaked increased transcription of PRCP, approximately 20% dead cells) (Figure 4B) and was significant when cells reached passage 27. Surprisingly, senescent cells were functionally active at working passages (P16–P19), for instance changes in PRCP, eNOS, and enhanced NO. The altered cell morphology was becoming increasingly evident following passage 27 (data are shown), highlighting a hallmark of aging. 

Senescence is a cellular response characterized apparently by numerous triggers including oxidative stress [38,39,40], telomere damage/shortening [41,42], mitochondrial dysfunction [39], and inflammation [40]. HPAECs are well correlated with a shortening of human telomerase reverse transcriptase (hTERT) length (Figure 4C), which decreased with increasing passages. As shown above, the buildup of PRCP mRNA reaches a high peak (passage 17) and begins to decline by cell passage 18 (Figure 2C). Interestingly, the decrease in the transcription of telomerase preceded the decrease in mRNA transcription of PRCP. Fibroblast growth factor 2 (FGF-2) was found to be responsible for cell survival and the formation of new blood vessels [43,44]. FGF-2 mimicked PRCP, peaking slightly earlier, but followed the same trend as PRCP, reinforcing the belief that it, too, promotes cell survival and the delay of endothelial cell senescence (Figure 4D). Together, these data demonstrated that the delayed loss of PRCP may be important in the delayed cell death, while its expression pattern may serve as an independent indicator for assessing cellular senescence. 

### 2.5. Effect of UM8190-Induced PRCP Inhibition on Mitochondrial Function

**Figure 5:**
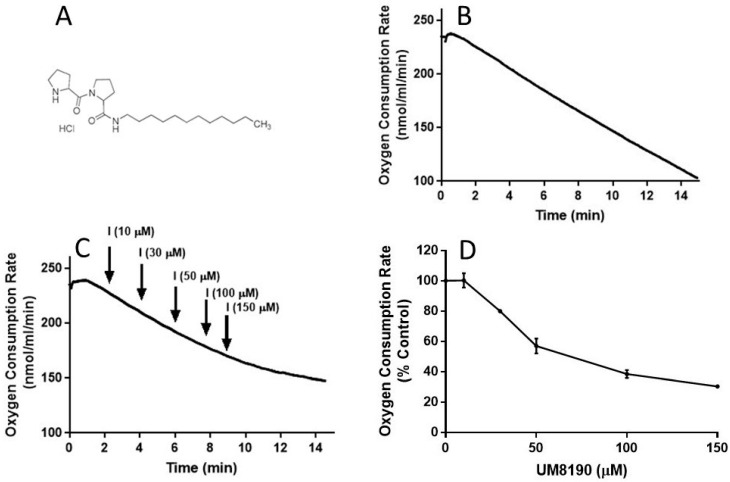
UM8190 treatment decreases oxygen consumption. (**A**), UM8190 structure. (**B**), Oxygen consumption of HPAECs as a result of mitochondrial activity was plotted in the absence of UM8190. Representative trace served as control. (**C**), After a basal measurement of ORC, measurement of OCR for HPAECs (5 × 10^5^ cells/mL) followed by the sequential addition of UM8190 (10–150 μM) with a measurement of OCR as indicated by arrows. OCRs were measured three times and plotted as a function of time. (**D**), The resulting UM8190 effects on OCR are plotted as a percentage of control. Data shown are the mean ± SEM. N = 3 of three independent experiments.

It is known that NO is a reversible inhibitor of the mitochondrial respiratory chain. We have hypothesized that this effect is also mediated by the activation of the PRCP-dependent pathway. To test this hypothesis, we determined the impact of UM8190 (Figure 5A), a selective inhibitor of PRCP, on mitochondrial respiratory function in both intact HPAECs and digitonin-permeabilized HPAECs, based on previously published guidance [45] with some modifications. 

To evaluate the integrity of cellular respiration, the respiratory rates of HPAECs were initially determined in 1 mL respiratory medium (bicarbonate free DMEM/F12 medium) with continuous stirring at 37 °C according to a previously described report [46,47] (Figure 5). The rate of O_2_ consumption linearly decreased (Figure 5B), suggesting that mitochondria are functional in the untreated HPAE cell line. 

NO inhibits mitochondrial complex I [48], which is discussed in detail elsewhere [49]. Next, studies were carried out to determine whether UM8190-induced PRCP inhibition could dose-dependently protect against NO-induced mitochondrial complex I inhibition. UM8190 is an inhibitor of PRCP (apparent *Ki* = 43 μM) [50]. Contrary to our hypothesis, treatment of HPAECs with UM8190, at pharmacological concentrations (10–150 μM), resulted in a reduction in mitochondrial O_2_ consumption rate (OCR) in the HPAE cell line (Figure 5C). The resulting UM8190 effects on OCR are plotted as a percentage of the control (Figure 5D).

Low concentrations of digitonin have been used to selectively permeabilize the plasma membrane of various cell lines [51] with no apparent effect on the nucleus [52]. UM8190-induced PRCP inhibition blocked mitochondrial function at >20 μM. 

**Figure 6:**
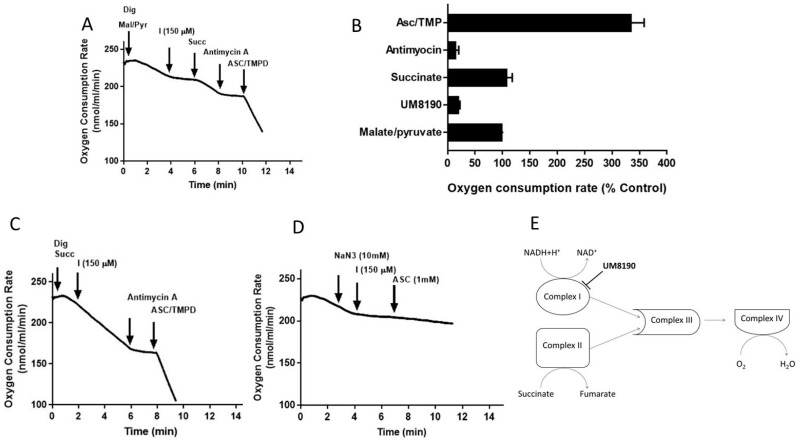
Effect of UM8190 on the respiration of digitonin-permeabilized HPAE cells. (**A**), Effect of UM8190 on Complex I. (**B**), Oxygen consumption rate as a percentage of control. (**C**), The inhibitory effect of UM8190 on Complex II. (**D**), Effect of UM8190 on cytochrome C oxidase. (**E**), A schematic diagram summarizes the effects of UM8190 on complexes of mitochondrial respiratory chain. Data are representative traces from three independent experiments.

Addition of digitonin (12 μM) did not alter the rate of basal respiration of HPAE cell suspension, suggesting that digitonin permeabilization maintains mitochondrial function. Next, investigations were performed to determine the effects of UM8190 on various complexes of mitochondria in the presence of digitonin-permeabilized HPAECs. Malate/pyruvate (5 mM), a substrate of complex I, stimulated an increase in oxygen consumption. The subsequent addition of UM8190 (150 μM) inhibited NADH oxidation leading to reduced oxygen consumption (Figure 6A). Succinate is a known substrate of complex II (succinate/quinone oxidoreductase) [53]. Succinate (5mM) addition restored oxygen consumption. However, respiration was inhibited by antimycin A (an inhibitor of complex III, 1 μM). Next, we examined the effect of N,N,N’,N’-tetramethyl-p-phenylenediamine (TMPD, 0.2 mM, an artificial electron donor) on complex IV in the presence of ascorbate (5 mM), which maintains TMPD in a reduced state. The addition of these to the respiratory medium rescued the antimycin A effect and the integrity of the mitochondria (Figure 6A). The data are summarized by OCR (Figure 6B). Because succinate bypassed the UM8190-induced PRCP inhibition effect, electrons entered through QH2, resuming oxidation. 

Like complex I, complex II generates ROS [54]. To assess whether UM8190 (150 μM)-induced PRCP inhibition would affect succinate oxidation, digitonin-treated HPAE cells were incubated with succinate (5 mM) (Figure 6C). While UM8190-induced PRCP inhibition (150 μM) was ineffective and antimycin A stopped oxidation, it shows that UM8190 is neither an inhibitor of complex II nor complex III of mitochondria. The addition of TMPD and ascorbate rescued the respiration of mitochondria, indicating the mitochondrial integrity is intact. Taken together, UM8190 was ineffective in inhibiting complex II (Figure 6C).

Sodium azide (NaN3) is an inhibitor of cytochrome C oxidase (complex IV). The addition of sodium azide (NaN_3_, 10 mM) to the non-permeabilized HPAE cell suspension rapidly inhibited the mitochondrial respiration rate and the addition of UM8190 did not rescue the NaN_3_ effect, which blocks cytochrome C oxidase (Figure 6D). While ROS production sites in mitochondria are complex I, complex II, and complex III, the scheme shown in Figure 6E summarizes that UM8190 inhibits complex I. These data indicated that UM8190-induced PRCP inhibition may inhibit the accumulation of potentially damaging levels of ROS.

### 2.6. Effect of UM8190 on Mitochondrial Generation of Reactive Oxygen Species (ROS)

**Figure 7:**
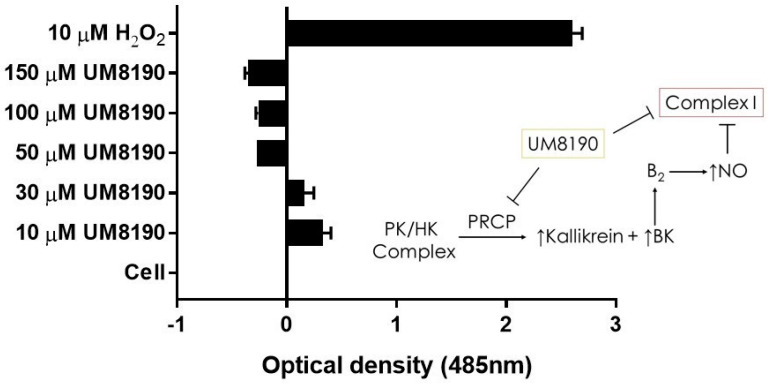
UM8190 compound inhibits mitochondrial Complex I ROS production. All the values are expressed as mean standard error of the mean of at least three independent experiments. Inset, a schematic diagram showing the proposed mechanism by which UM8190 exerts its effects on the HPAECs including the kallikrein–kinin forming pathway and mitochondrial complex I.

Complex I, as a major ROS production site, plays an important role during normoxic condition [16]. UM8190-induced PRCP inhibition blocked the mitochondrial complex I. CM-H2DCFDA, an indicator for ROS in cells, has been used to detect the level of ROS in endothelial cells [55]. Briefly, it detects intracellular superoxide radical anion levels in live cells at Ex/Em: 485/530 nm [56]. Investigations were performed to determine the effect of UM8190-induced PRCP inhibition on CM-H2DCFDA in working passage (P10, 10% of growing cells scored as β-Gal positive) HPAECs by mitochondria. To avoid a non-specific product, we used the lowest CM-H2DCFDA concentration [57]. Hydrogen peroxide (H_2_O_2_) was used as the positive control. The cells were treated with different concentrations of UM8190 (10–150 μM) and H_2_O_2_ (10 μM) for 19 h at 37 °C incubator with 5% CO_2_. At the end of the incubation, the cells were washed two times with 1× PBS followed by treatment of CM-H2DCFDA (5 μM) for 1 h at 37 °C incubator with 5% CO_2_. Following the incubation, CM-H2DCFDA solution was removed, and the cells were washed with PBS to remove free dye, then 100 μL of HBSS was added to each well and read at 485 nm in a BioTek Synergy 2 plate reader (Agilent Technologies, Santa Clara, CA, USA)) (Figure 7). Compared to H_2_O_2_-treated cells, UM8190-induced PRCP inhibition dose-dependently showed the generation of ROS at two concentrations (10 μM, 30 μM), but protected the cells at higher concentrations, suggesting that the inhibition of PRCP results in an accumulation of ROS at a low concentration (Figure 7). 

However, this ratio was significantly inhibited by concentrations above 30 μM UM8190. Currently, we are unable to provide a readily available explanation for this finding. One possible explanation for the decrease in ROS formation is that UM8190 directly blocks complex I, which could be potentially clinically useful. The schematic diagram to the right shows that the activation of the B_2_ receptor by PRCP-dependent PK activation leads to bradykinin-mediated NO formation and inhibition of complex I. It appears that UM8190 inhibits both PRCP-dependent PK activation and complex I. This new finding will provide a new lead compound and target for further in vitro and in vivo studies in mitochondria.

## 3. Discussion

PRCP has emerged as a cardioprotective protease with imperative implications for cardiovascular health. PRCP may have crucial physiological functions in the kidney, heart, brain, and in particular, in endothelial cells, which regulate in coordination with vascular smooth muscle cells to help the blood flow to tissues. Recent research studies provided compelling evidence that PRCP protects against the impairment of both the heart [18] and the kidney [58] and helps to dampen elevated blood pressure [17]. PRCP is associated with a significantly increased risk of preeclampsia [28], metabolic condition [13], IgA nephropathy (IgAN), and autoimmune disease pathogenesis [59]; however, several of the underlying mechanisms have yet to be fully elucidated. 

The aim of the present study was to examine the effects of aging on PRCP in cultured HPAE cells. Due to their ability to regulate various vasoactive peptides that help to control water and electrolytes, cause smooth muscles in the heart and the blood vessels to relax, and delay thrombus formation through the activation of the plasma kallikrein–kinin system (KKS) and regulation of angiotensin molecules, PRCP-dependent pathways appear to play a remarkable role in vascular physiology and in the development of cardiovascular disease. The major findings of this study are as follows: (1) this study addressed a significant gap in PRCP data related to endothelial injury during the aging process; (2) as the cell ages, the expression of PRCP and the activity of PK, a PRCP substrate, are increased in the early stages of senescent cells; (3) the possible molecular mechanisms underlying PRCP-induced vascular protection are found to be via a progressive elevation of the production of NO during the replicative cell senescence; (4) increased PRCP expression is associated with progressively reduced hTERT expression in age-related HPAEC dysfunction, suggesting PRCP could be an early predictor of endothelial dysfunction; and (5) UM8190 may also act as an inhibitor of complex I of the mitochondrial respiratory chain.

Both plasma KKS and the renin–angiotensin system (RAS) serve a central role in the regulation of renal, cardiac, and vascular physiology. The activation of these two intertwined pathways plays a significant role in numerous common pathology conditions including inflammation, heart failure, renal disease, and diabetes. While both KKS and RAS are tightly regulated systems and recognize different receptors that are expressed on endothelial cells, they keep their effects at just the right level by sharing enzymes (angiotensin-converting enzyme, PRCP) that perform more than one function. While kinins (BK, des-Arg^9^-bradykinin), metabolites of both plasma KKS and tissue KKS, can increase vascular permeability and vasodilation, angiotensin II and angiotensin III molecules, metabolites of RAS, can cause vasoconstriction in vivo. The endothelium maintains the delicate balance between these two physiological functions [60] to provide instantaneous power to generate the forward-moving pushing blood wave in order to prevent coagulation, fibrinolysis, and inflammation by producing NO and other regulatory factors [61], while accepting nutrients from the blood. 

The process of vascular disease is complex and is co-regulated by multiple interwoven signaling pathways leading to endothelial dysfunction or damage, whereas increasing evidence suggests that chronic low-grade inflammation in the pathology of numerous age-related chronic conditions (such as insulin resistance, hypertension, vascular aging) is one of the initiating events [62,63,64]. Endothelial senescence is considered to be a hallmark of aging [65]. Most importantly, the impairment of the endothelium contributes to compromised tissue perfusion and induces functional decline in older individuals [66].

By investigating the effects of the HK and PK complex on senescent endothelium cells, we found a great starting point to investigate whether the PK activation in the presence of cell-bound HK may help to delay cell dysfunction. Evidence has revealed the interplay of certain facets of autophagy and PRCP under physiological conditions [67], and a causal relationship was found among kallikrein activity, angiotensin II, and PRCP [18,68]. This study showed that the PRCP-mediated kallikrein–kinin pathway delayed endothelial cell senescence by promoting cellular survival through generation of NO, a vasodilative molecule. Taken together, this finding may be explained by the idea that PRCP helps to prevent free radical-induced cell injury via not only the production of NO, but also through a previously described process of autophagy [67], a complex and diverse homeostatic phenomenon.

Cellular replicative senescence is a slow biological process of aging that involves the accumulation of various changes to the internal environment of a cell, most notably the buildup of acidic β-gal. These changes, both structural and molecular in nature, disable metabolism of many cellular processes and eventually lead to the induction of apoptosis. As cells divide, the ends of chromosomes, and telomeres, slowly shorten. Once telomere shortening reaches critical levels, those chromosomes may no longer replicate properly, leading to cellular process complications and apoptosis. Telomerase is a reverse transcriptase that is responsible for counteracting telomere shortening and prolonging cellular life span. To explore whether PRCP possesses a novel function in a pro-survival pathway, we compared its expression against the expression pattern of hTERT. PRCP serves as a focal point in response to a variety of extracellular stimuli including Ang II, Ang III, kinin, and the plasma HK–PK complex. One of the crucial findings of this study demonstrated that the expression and function of PRCP was age-dependent. We also found that in comparison with low hTERT expression, PRCP expression was elevated in HPAECs. Since our study found an age-dependent increase in PRCP expression that was proportional to the decrease in telomerase expression, monitoring PRCP activity may be predictive of biological age. 

Prior studies have shown that the endothelial senescence is triggered by numerous senescence stressors including oxidative stress and mitochondrial dysfunction [36,69]. In a previous work, we showed that overexpression of PRCP enhanced certain markers of mitochondrial autophagy [67], the process by which damaged mitochondria are removed under mitochondrial toxicity conditions [70], or may help to delay cell death. The chronic defects in mitochondrial proton-pumping NADH/ubiquinone oxidoreductase (complex I) [71] or metformin-induced inhibition of complex I [72] with an IC_50_ value of 20 mM [73] suppressed basal autophagy [71] and prevented ROS generation by complex I. 

The kallikrein proteomics analysis of the diabetic macular edema [74] and antibody-array interaction mapping method to detect the activation of the plasma PK [75], and hydrolysis of PK to kallikrein when bound to HK on cells to liberate BK [76], have demonstrated their crucial role involved in disease-related signaling networks. Multiple lines of evidence indicate that damage occurring to the vascular endothelium is an early event in cardiovascular disease, switching from anti-thrombotic properties to a prothrombotic state, and may therefore play a key pathogenic role in the disease. Ang II display numerous physiological actions, related mainly to the regulation of electrolytes and fluid osmolarity. While chronic elevation of Ang II induces hypertension that is accompanied by enhanced thrombosis in arterioles through the angiotensin type 2 receptor-dependent pathway [77], treatment with angiotensin-converting enzyme inhibitors and angiotensin II type 1 receptor antagonists appears to affect the balance between the RAS and KKS axis, regulating not only blood pressure but also thrombosis [78]. In contrast, prekallikrein null mice (*Klkb1*^−/−^) exhibited delayed artery occlusion times on the rose Bengal and ferric chloride thrombosis models [79]. Interestingly, ablation of *Klkb1* dampened the progression of atherosclerosis in mice on an Apoe-deficient background [80]. We also showed that B_2_ receptor knockout mice were protected from thrombosis by increased NO and prostacyclin [81]. In the brain, compelling evidence demonstrated that the inhibition of plasma kallikrein reduced matrix metalloproteinase-9 activity [82] following stroke and tissue plasminogen activator (tPA) therapy. Thus, PK activation appears to be tissue-specific. Remarkably, BK has been recognized as an inducer of tPA [83,84]. 

UM8190 inhibits PRCP [50]. It also appears to inhibit complex I of the mitochondrial respiratory chain. Evidence indicates that complex I inhibitors are capable of improving glucose homeostasis [85]. Thus, while the clinical safety of UM8190 compound is largely unproven, its effect on complex I offers a promising and exciting strategy to control hyperglycemia and help prevent diabetes complications. Together, these data indicated that PRCP-dependent PK activation may serve as cell signaling molecule for a normal biologic process. Moreover, PRCP expression is elevated when endothelial cells are activated and may provide a novel strategy for the prediction of the risk and severity of vascular disease.

## 4. Materials and Methods

### 4.1. Materials

The HPAECs, EGM, FBS growth supplement, HEPES, 0.25% Trypsin-EDTA, and trypsin-neutralizing solution were used as supplied by Clonetics (San Diego, CA, USA). The HK and PK were purchased from Enzyme Research Laboratory (South Bend, IN, USA). S2302 was purchased from DiaPharma (Franklin, OH, USA). The primary antibody, Goat Anti-Human PRCP, and the secondary Anti-Goat IgG: Whole Molecule, Peroxidase Conjugate were purchased from Bioscience (Long Beach, CA, USA). Mouse Anti-Human β-Actin was purchased from Santa Cruz Biotechnology (Santa Cruz, CA, USA). The “Nitrate/Nitrite Fluorometric Assay Kit” was purchased from Cayman Chemicals (Ann Arbor, MI, USA). The Cellular Senescence Assay Kit was purchased from Chemicon International (Temecula, CA, USA). TRIzol, Super Signal West Femto Maximum Sensitivity Substrate, RiPA Buffer, Super Signal West Femto Maximum Sensitivity Substrate were supplied by ThermoFisher Scientific (Rockford, IL, USA). The Precision Plus Protein standard (Dual Color) was purchased from Bio-Rad (Rockford, IL, USA). Ethidium bromide was from Sigma-Aldrich (St. Louis, MO, USA).

### 4.2. Endothelial Cell Culture

Human pulmonary artery endothelial cells (HPAECs) were grown and subcultured over time, from Passage 7 to Passage 40 in endothelial growth medium (EGM) according to manufacturer recommendations. Cells were allowed three days to proliferate before each subculture procedure using 0.25% Trypsin-EDTA, DPSB, and the trypsin-neutralizing solution (TNS). Right before detaching the cells from the flask to be subcultured, condition medium was removed and aliquoted and saved for further study. The cells were grown in fetal bovine serum (FBS) supplemented with EGM at a constant temperature of 37 °C, in an environment with a constant carbon dioxide level of 5%. 

### 4.3. HK/PK Activity

After obtaining a cell pellet from the trypsinized flask, 100 µL of the HPAECs were seeded at 30,000 cells/well of the 96-well plate and incubated at 37 °C incubator with 5% CO_2_. The cells were washed with HEPES–NaHCO_3_ buffer (137 mM NaCl; 3 mM KCl; 14.7 mM HEPES; 1 mM MgCl_2_; 2 mM CaCl_2_; 5.5 mM glucose and 0.1% gelatin, pH 7.1) and blocked with 1% HEPES–gelatin mixture for an additional 1 h to reduce any non-specific binding. At the end of incubation, the cells were washed twice with HEPES and treated with 20 nM HK for one hour. At the end of the incubation, the cells were washed and treated with 20 nM PK and incubated for an additional hour. Finally, the cells were treated with 0.5 mM a HD-Pro-Phe-Arg-paranitroaniline substrate (S2302) and incubated for an hour. All incubations took place at a constant temperature of 37 °C incubator with 5% CO_2_. The activity of formed plasma kallikrein was quantified by detecting the amount of free paranitroanilide in vitro, at 405 nm, using the BioTek ELX800 Plate Reader (Cole-Parmer, Vernon Hills, USA).

### 4.4. Western Blot

Adherent cell monolayers were washed with DPSB. The protein cell lysates were extracted using the 1× RIPA Buffer (Thermo Fisher Scientific, Rockford, IL, USA) containing protease inhibitors and separated by electrophoresis in a 10% SDS-PAGE. After electrophoresis, the gel was transferred to a nitrocellulose membrane using electro-blot buffer (20% methanol, in 1× Tris/glycine) at 4 °C for one hour. Once transferred, the membrane was blocked with 5% non-fat dry milk in PBS containing 0.1% Tween-20 (PBST) for an hour at room temperature. Then, the membrane was treated overnight at 4 °C with 1:20 dilution of the primary antibody (goat anti-human PRCP from Bioscience) and goat anti-human β-actin (1:100, Santa Cruz, CA, USA). The next day, the membrane was washed thrice with PBST and then treated with 1:1000 dilution of secondary antibody (anti-goat IgG: whole molecule, peroxidase conjugate from Bioscience). The membrane was washed thrice again with PBST before being treated with the “Super Signal West Femto Maximum Sensitivity Substrate,” courtesy of Thermo Scientific (Rockford, IL, USA), a chemiluminescent substrate to be used for imaging with the ChemiDoc Imager (Bio-Rad, Hercules, CA, USA). 

If the membrane was treated merely with goat anti-human PRCP, the membrane was washed with 1× PBS and stripped with Thermo Scientific’s “Restore Western Blot Stripping Buffer” at room temperature for 15 min before being blocked again and re-probed with 1:100 dilution of the primary antibody (mouse anti-human β-Actin) from Santa Cruz Biotechnology (Santa Cruz, CA, USA), followed by 1:1000 dilution of secondary antibody (anti-goat IgG or anti-mouse IgG: whole molecule, peroxidase conjugate). After blocking, antibody treatments, and washing, the membrane was imaged again and a ratio between the two densities was calculated.

### 4.5. Nitrite/Nitrate Assay

The condition medium which was collected and stored over the course of growing HPAECs passages was used to measure nitric oxide. The “Nitrate/Nitrite Fluorometric Assay Kit” (Cayman Chemicals) was used to quantify the metabolites of endothelial nitric oxide. Samples of condition medium were adjusted to 80 µL with a 50/50 mix of fresh growth medium and assay buffer. Enzyme cofactors and nitrate reductase were added to each well and the plate was incubated for an hour at room temperature. Afterwards, DAN (2,3-diaminoaphthalene) was added, followed by sodium hydroxide. The plate was read (Ex 360–365 nm, Em 430 nm). By using the standard curve, the concentration of Nitrate + Nitrite of the sample was calculated.

### 4.6. Cellular Senescence Assay Kit 

The “Chemicon International” Cellular Senescence Assay Kit was used on various passages of growing HPAE cells to qualify their percentage of senescent cells. Senescent-associated β-galactosidase (SA-β-gal) is only present in senescent cells, and Chemicon’s kit provides all reagents needed to detect SA-β-gal activity at pH 6.0 in cell cultures. SA-β-gal catalyzes the hydrolysis of X-gal, causing the accumulation of the blue dye in senescent cells. Detection and quantification of β-gal-positive cells were based on the protocol provided in SA-b-gal-positive cell detection kit (Chemicon’s kit) and a previously established method and approach [86]. Briefly, the cells were examined on a light microscope with an attached camera using a 10× objective. The β-gal-positive cells were blue. The fraction of β-gal-positive cells was determined by counting the number of blue cells in 5 non-overlapping images from various fields of view within each well. Cells were dislodged by trypsin-EDTA and counted to obtain total number of cells per condition. The β-gal-positive (blue) cell count was divided by the number of total cell count to calculate the percentage senescent cells. Representative images were used for figures.

### 4.7. Reverse-Transcriptase Polymerase Chain Reaction and Agarose Gel

Cell pellets were resuspended using TRIzol (Invitrogen-Life Technologies, Carlsbad, CA, USA). The mRNA was extracted from subcultured HPAEC using QIAGEN’s “RNase-Free DNase Set.” RNA was put through reverse-transcription via Invitrogen’s “SuperScript III One-Step RT-PCR (with Platinum Taq) Kit.” We bought multiple human DNA primers from Invitrogen, including hTERT (SENSE–5′ ATG GGG ACA TGG AGA ACA AG 3′ and ANTISENSE–5′ GTG AAC CTG CGG AAG ACG GT 3′), β-Actin (SENSE–5′ TGA ATG GAC AGC CAT CAT GGA C 3′ and ANTISENSE–5′ TCT CAA GTC AGT GTA CAG GAA AGC 3′), FGF-2 (SENSE–5′ TCA GCT CTT AGC AGA CAT TGG AAG AAA AAG 3′ and ANTISENSE–5′ GGA GTG TGT GCT AAC CGT TAC CTG GCT ATG 3′), PRCP (SENSE–5′ ATG GGC CGC CGA GCC CTC CTG 3′ and ANTISENSE–5′ GGT TGG TTG GCA AGT GTA GG 3′), and eNOS (SENSE–5′ ATG TTT GTC TGC GGC GAT GTT AC 3′ and ANTISENSE–5′ ATG CGG CTT GTC ACT TCC TG 3′). The PCR products were visualized using 1–2% agarose gel containing ethidium bromide (agarose powder and ethidium bromide were purchased from Invitrogen and Sigma, respectively) depending on the size of the expected PCR product. 

### 4.8. Oxygen Consumption Rate Measurements on HPAE Cells

Mitochondrial respiration is measured using Oxytherm Clarke-type electrode System (Hansatech, distributed by PP System, Amesbury, MA, USA) to monitor oxygen concentration in non-permeabilized or digitonin-permeabilized human pulmonary artery endothelial (HPAE) cells. 

The confluent HPAE cells were dissociated using trypsin-EDTA solution. Trypsinized cells were centrifuged at 180× *g* for 5 min at RT and the HPAE cell pellet was washed twice with DPBS and re-centrifuged. The final HPAE cell pellet was re-suspended in 1× PBS to a final cell density of 50 × 10^4^ cells/mL. 100 μL of cell suspension was transferred to the oxytherm chamber containing 900 μL of Sodium Carbonate-free DMEM/F/12 medium (Sigma, St. Louis, MO, USA) for each non-permeabilized assay (50 × 10^3^ cells per run). 

HPAECs’ mitochondrial respiration rate was measured in 1.0 mL of total respiration solution (Sodium Carbonate-free DMEM/F/12 medium) at 37 °C under continuous stirring. An amount of 100 μL of HPAE cells (50 × 10^4^ cells/mL) was added to equilibrated media in Oxytherm incubation chamber with constant stirring. The mitochondrial respiration was performed in the absence or presence of UM8190. 

To check the mechanistic mitochondrial function for each complex, respiration rate was measured either through complex I or complex II in mitochondria buffer (20 mM HEPES, 120 mM KCl, 2 mM KH_2_PO_4_, 2 mM MgCl_2_, 1 mM EGTA, pH = 7.3). We examined complex I function by using digitonin (12 μM)-permeabilized HPAE cells in mitochondria buffer and started the respiration through complex I by adding malate (5 mM) and pyruvate (5 mM). The respiration rate of malate/pyruvate was inhibited with 1 μM rotenone (a complex I inhibitor) and then the respiration was restored by adding succinate (5 mM) to stimulate mitochondrial respiration through complex II and generate ATP via OXPHOS (succinate dehydrogenase). The mitochondrial respiration, after addition of succinate, was inhibited by antimycin A (an inhibitor of coenzyme Q–cytochrome C reductase and complex III or cytochrome bc_1_). Finally, the integrity of mitochondria was checked by adding ascorbate (5 mM) and N,N,N,N-tetramethyl-p-phenylenediamine (TMPD; 0.2 mM; Sigma, St. Louis, MO, USA) to rescue respiration by stimulation of cytochrome C oxidase (complex IV). Respiration rates were measured for at least 15 min in each step or until a steady state was reached. Baseline was recorded for normalization, then the test compounds were added alone or in combination. To examine the mitochondrial respiration rate through complex II, we used digitonin (12 μM)-permeabilized HPAE cells in mitochondria buffer and added succinate (5 mM) to start respiration through complex II. The respiration was inhibited by antimycin A (an inhibitor of coenzyme Q–cytochrome C reductase and complex III or cytochrome bc_1_). Finally, the integrity of mitochondria was checked by adding ascorbate (5 mM) and N,N,N,N-tetramethyl-p-phenylenediamine (TMPD; 0.2 mM) to rescue respiration by stimulation of cytochrome C oxidase (complex IV).

### 4.9. Statistical Analysis

All data are expressed as the mean ± SEM. Parametric data were analyzed using Student’s *t*-test for significant difference. Statistical analyses were performed using GraphPad Prism software, version 7.

## 5. Conclusions

In summary, the evidence presented herein indicates an actively protective role for the PRCP-dependent pathway against oxidative stress in the age-related endothelial cell senescence. This study provides a link between dysfunctional (i.e., excessively short) telomeres and the generation of PRCP. PRCP may thus be a highly promising diagnostic marker for the visualization of CVD. While the role of senescent cells has been confirmed in numerous age-related diseases including insulin resistance [87] and macular degeneration [88], inflammation-induced ROS production [89] is implicated in aging. Our data may provide a novel strategy for the prediction of vascular disease and further implicate mitochondria which are critical disease drivers when metabolically dysfunctional [90]. In particular, our studies provided new insights into the mechanism of action of UM8190 beyond age-related disease states. The findings of this study facilitate the future studies attempting to characterize the functional consequences of UM8190 on mitochondria. For instance, UM8190 (along with other UM8190 analogs) may be used to target oxidative phosphorylation and mitochondria-related metabolism to prevent metastasis and the progression of cancers, raising the possibility of it being used in the future as a chemotherapeutics agent. Testing UM8190 and its analogs will shed light on the clinical applications of these promising therapeutics.

## Data Availability

The original contributions from this study are included in the article; further inquiries can be directed to the corresponding author.

## Supplementary Materials

The following supporting information can be downloaded at: https://www.mdpi.com/article/10.3390/molecules29102219/s1, Figure S1: Immunoblot analysis of PRCP protein expression in HPAE cells.

## Abbreviations

Ang III	Angiotensin III
Ang 2–7	Angiotensin 2–7
ATP	Adenosine triphosphate
β-Gal	β-galactosidase
BKB_2_	Bradykinin B2 receptor
BK	Bradykinin
CVD	Cardiovascular diseases
EGFR	Epidermal Growth Factor Receptor
eNOS	Endothelial nitric oxide synthase
GAPDH	Glyceraldehyde-3-phosphate dehydrogenase
H_2_O_2_	Hydrogen Peroxide
HK	High molecular weight kininogen
HPAEC	Human pulmonary artery endothelial cells
hTERT	Human telomerase reverse transcriptase
IgAN	IgA nephropathy
KKS	Kallikrein–kinin system
mDNA	Mitochondrial DNA
Mal/Pyr	Malate/pyruvate
NAD	Nicotinamide Adenine Dinucleotide
NADH	Nicotinamide Adenine Dinucleotide
NADPH	Nicotinamide adenine dinucleotide phosphate
NaN_3_	Sodium azide
NOOCR	Nitric oxideMitochondrial O_2_ consumption rate
PAR	Protease-activated receptor
PK	Prekallikrein
PRCP	Prolylcarboxypeptidase
PTCA	Percutaneous transluminal coronary angioplasty
RAS	Renin–angiotensin system
ROS	Reactive oxygen species
SHRs	Spontaneously hypertensive rats
T2DM	Type 2 diabetes mellitus
TMPD	N,N,N′,N′-tetramethyl-p-phenylenediamine
