# Supplementary material for: Increased Prolylcarboxypeptidase Expression Can Serve as a Biomarker of Senescence in Culture
Source: Molecules. 2024 May 9;29(10):2219. doi: 10.3390/molecules29102219 (PMC11123917; doi:10.3390/molecules29102219)
Supplement: Supplementary file 1 [file molecules-29-02219-s001.zip › molecules-2961545-supplementary.pdf]

Supplementary Material File S1. Western blot

Figure 2A original blot

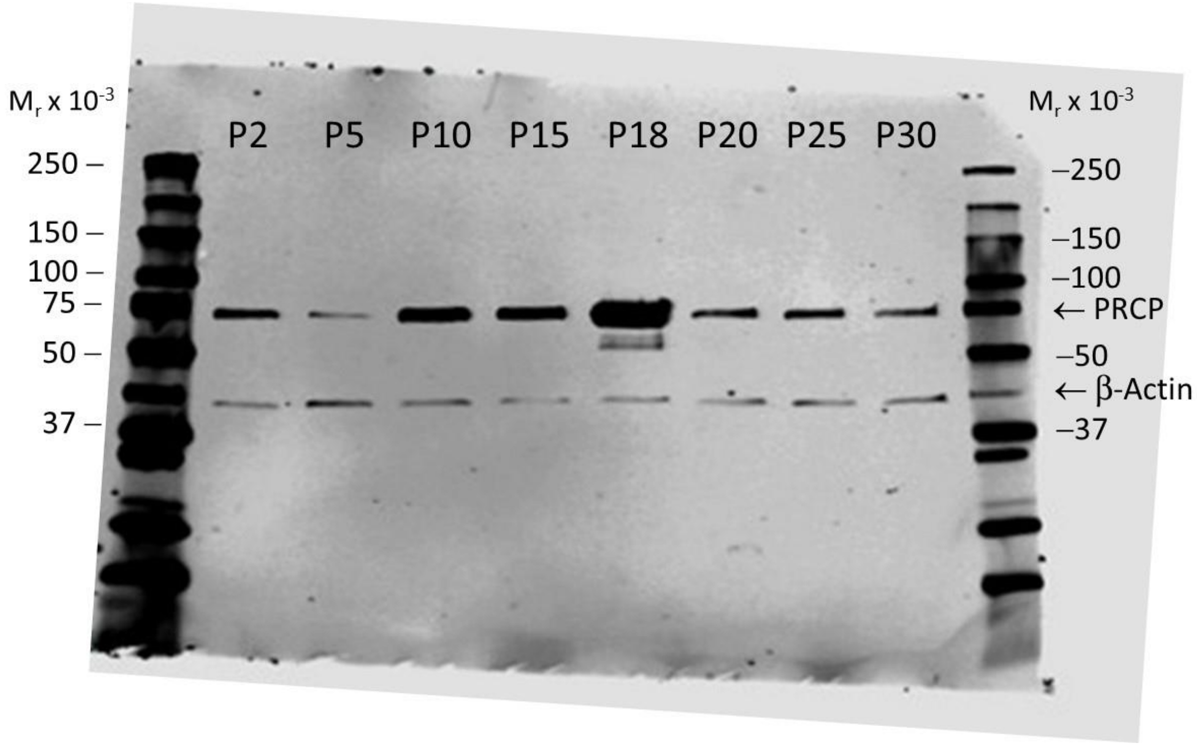

Figure S1. **Immunoblot analysis of PRCP protein expression in various HPAE cell passages (P2-P30)**. HPAECs were homogenized in lysis buffer containing protein inhibitor cocktail. Lysates (100 μg/lane) were resolved on 4-20% SDS-PAGE and then transferred on to nitrocellulose membrane. β-Actin was used as endogenous control. PRCP and β-actin were detected by a conventional chemiluminescent method using anti-PRCP and anti-β-actin antibodies.
